# Impact of Central Line-Associated Bloodstream Infections on Mortality and Hospital Stay in Adult Patients at a Tertiary Care Institution in Cali, Colombia, 2015–2018

**DOI:** 10.3390/jcm13185376

**Published:** 2024-09-11

**Authors:** Jorge Mario Angulo Mosquera, Jorge Karim Assis Reveiz, Lena Barrera, Yamil Liscano

**Affiliations:** 1 Grupo de Investigación en Salud Integral (GISI), Department of Health, Universidad Santiago de Cali, Cali 760035, Colombia; 2 Department of Research and Education, Clínica de Occidente S.A., Cali 760044, Colombia; ophthalmology2013@gmail.com; 3 Research Group CEDETES, School of Public Health, Faculty of Health, Universidad del Valle, Cali 760032, Colombia; lena.i.barrera@correounivalle.edu.co

**Keywords:** central line-associated bloodstream infections, mortality, hospital stay, central vascular catheter, tertiary care, resistant microorganisms

## Abstract

**Background:** Central line-associated bloodstream infections (CLABSIs) are a significant healthcare challenge globally, increasing mortality risk and complicating central vascular catheter use. In Colombia, few studies have assessed the impact of CLABSIs on hospital stay and mortality. **Objective:** To determine the association between CLABSIs and discharge outcomes and hospital stay duration in adult patients at a tertiary care institution in Cali, Colombia, from 1 January 2015 to 31 December 2018. **Methods:** A nested case–control study was conducted. The odds of mortality associated with CLABSIs were estimated using conditional logistic regression. Non-conditional logistic regression was used to determine the odds of mortality when CLABSIs were caused by resistant microorganisms. Hospital stay duration, catheter duration, and time from catheter insertion to discharge were compared between patients with and without CLABSIs. The most frequent etiological agents were identified. **Results:** Patients with CLABSIs had 3.89 times the odds of mortality (95% CI [1.33–11.31], *p* = 0.013) compared to those without CLABSIs. The odds of mortality for patients with resistant microorganism CLABSIs were 4.04 times (95% CI [1.17–13.96], *p* = 0.027) higher than those with sensitive microorganism CLABSIs. Hospital stay duration (median = 51 days vs. 17 days; *p* = 0.000), catheter duration (median = 19 days vs. 7 days; *p* < 0.001), and time from catheter insertion to discharge (median = 40 days vs. 9 days; *p* < 0.001) were significantly longer in CLABSI patients. *Klebsiella pneumoniae* was the most isolated pathogen (20.2%), followed by *Staphylococcus aureus* (14.9%). **Implications:** CLABSI patients have longer catheter and hospitalization durations and higher mortality risk. Resistant microorganism CLABSIs are associated with elevated mortality risk. **Conclusions:** This study corroborates the positive relation between CLABSI and the mortality risk, which is influenced by resistant bacteria, though causality is not established. CLABSI is also linked to longer hospital stays, underscoring the need for improving infection control strategies

## 1. Introduction

Central line-associated bloodstream infections (CLABSIs) are among the most significant healthcare-associated infections (HAIs) globally. These infections greatly impact patient outcomes, hospital stays, and healthcare costs, posing severe complications for the use of central line (CL). Given the essential role of CL in medical treatments, addressing the associated risks of CLABSIs is crucial to improving patient safety and healthcare quality [1,2]. Approximately 15–30% of nosocomial bacteremias are related to vascular catheters, with an attributable mortality rate of 12–25% [3,4,5]. These statistics underscore the urgent need for effective preventive measures and treatment strategies for CLABSIs.

Studies have shown that patients with CLABSIs have a 2.71 times higher risk of mortality and an extended hospitalization duration of approximately 20 days [6,7]. The development of CLABSIs adversely affects patient prognosis, making it imperative for healthcare providers to ensure high-quality care to prevent such infections. For instance, Guerrero-Diaz et al., 2024 [8] highlighted the well-documented adverse outcomes of CLABSIs, such as increased antibiotic use, longer hospital stays, and higher healthcare costs. Their study noted a significant reduction in the CLABSI rate from 5.71 to 1.26 per 1000 catheter days over the study period, attributed to improved adherence to the Aseptic Non-Touch Technique (ANTT) and effective implementation of quality improvement strategies.

Further research by Alshahrani et al., 2023 [9] reported that the mean hospital stay before CLABSI occurrence was 16 ± 13.3 days, significantly longer than the non-ICU length of stay. The most commonly isolated pathogen was Enterococcus spp., with frequent use of peripherally inserted central catheters (PICC) lines. Additionally, lower respiratory infections were the most burdensome infectious syndrome, and pathogens like *Escherichia coli*, *Staphylococcus aureus*, and *Klebsiella pneumoniae* were among the leading contributors to AMR-related deaths [10]. Significant independent risk factors for ICU-acquired CLABSI included the use of a double-lumen catheter, CL insertion before 2011, and prolonged CL exposure. Additionally, another systematic review noted a pooled CLABSI occurrence rate of approximately 8% in cancer patients, with various risk factors identified [9].

In a multinational prospective cohort study, Rosenthal et al., 2023 [11] found that key risk factors for CLABSIs included longer hospital stays, increased central line days, public hospital settings, and medical–surgical ICUs. Femoral and internal jugular central lines posed higher risks, whereas PICCs were not associated with increased risk. This study suggests focusing on reducing hospital and central line days, using PICCs over riskier central line sites, and adhering to evidence-based CLABSI prevention practices.

In Colombia, the necessity to monitor device-associated infections (DAIs) was recognized in 2010. By 2012, scientific evidence highlighted the magnitude of catheter-associated bloodstream infections (CA-BSIs), identifying them as the most frequent type of DAI [12]. The National Health Quality Report of 2015 documented 4485 cases of DAIs, with 1749 attributed to CA-BSIs, accounting for 39% of all reported cases. The incidence rate for CA-BSIs was 3.2 per 1000 central catheter days [13]. These figures emphasize the significant burden CA-BSIs place on the Colombian healthcare system, highlighting the need for effective infection control practices and continuous surveillance.

Álvarez-Moreno et al., 2016 [14] conducted a multicenter study to analyze the impact of a multidimensional International Nosocomial Infection Control Consortium (INICC) approach on CLABSI rates. The study included 2564 patients in four adult ICUs and 424 patients in two pediatric ICUs across four hospitals in two Colombian cities from June 2003 to April 2010. The baseline CLABSI rate of 12.9 per 1000 central line (CL) days was reduced to 3.5 per 1000 CL days after implementing the INICC approach, representing a 73% reduction in the CLABSI rate (relative risk, 0.27; 95% CI, 0.14–0.52; *p* = 0.002). This highlights the effectiveness of a multidimensional infection control approach in significantly reducing CLABSI rates in Colombian ICUs.

Current research on CLABSIs has provided valuable insights into their prevalence, risk factors, and outcomes. However, further investigation is necessary, particularly in regions like Colombia, where data are limited. Understanding regional variations and specific challenges in managing CLABSIs can help tailor interventions to be more effective and context-specific. The proactive approach taken by Colombian healthcare institutions to monitor and report DAIs is commendable, but more comprehensive studies are needed to fully understand the impact of CLABSIs on patient outcomes.

This study aims to determine the association between CLABSIs and both discharge outcomes and hospital stay duration in adult patients at a tertiary care institution in Cali, Colombia, from 1 January 2015 to 31 December 2018. By enhancing understanding of CLABSIs, this study will guide effective interventions to improve patient care and reduce healthcare-associated complications. This knowledge is crucial for guiding policy decisions and improving clinical practices to prevent these infections, ultimately contributing to better healthcare outcomes in Colombia and similar contexts.

## 2. Materials and Methods

### 2.1. Type of Study

A nested case–control study within a cohort was conducted on individuals admitted to the institution between 1 January 2015 and 31 December 2018 with a CL other than for dialysis. The cohort was matched 2 to 1 based on the Charlson index, age, and catheter insertion date for non-exposed individuals versus the CLABSI diagnosis date for exposed individuals.

Cases included patients who died for any cause within the cohort. The study took place in a tertiary and quaternary care institution, using records of CLABSI and medical histories. For the cohort, we measured the following durations: catheter duration from insertion to removal or discharge, hospitalization length post-catheter insertion, and total hospitalization duration.

In the case–control analysis, the outcome variable ‘status at discharge’ (alive or dead) was determined based on the presence of CLABSI. Data on causative microorganisms (etiological agent and resistance profile) were collected. Microorganisms were considered resistant if they exhibited phenotypic resistance to specific classes of antibiotics aligned with established resistance mechanisms. For Gram-negative bacteria, resistance was defined by the presence of Extended-Spectrum Beta-Lactamases (ESBLs), which confer resistance to third- and fourth-generation cephalosporins, and Carbapenemase-Producing Enterobacteriaceae (CPE), which inactivate carbapenems. For Gram-positive bacteria, resistance was defined by resistance mechanisms such as the production of Methicillin-Resistant Staphylococcus aureus (MRSA) and vancomycin-resistant Enterococci (VRE), which resist beta-lactams, glycopeptides, lipopeptides (e.g., daptomycin), and oxazolidinones (e.g., linezolid) [15,16,17].

The cohort was formed with those who were with central line catheters from 1 January 2015 to 31 December 2018. Patients who died (cases) were matched with those discharged alive based on hospitalization dates within six months, age (±5 years), and Charlson index categories (0–1, 2–3, 4+). Matching was performed at a ratio of three controls per case, with some groups having one or two controls per case.

This study included all patients with CLABSI, considering only the first event per patient and one CL per patient identified by the institution’s surveillance system. For non-exposed patients, only two homologous patients per CLABSI patient were included.

### 2.2. Definition of Exposure

- Catheter-Associated Bloodstream Infection 1: This is a laboratory-confirmed bloodstream infection where a pathogenic organism is identified, and a central line was present during the dates of the laboratory-confirmed bacteremia or the day before the bacteremia [18,19];
- Catheter-Associated Bloodstream Infection 2: This requires the patient to have a fever, hypotension, or chills, and the same commensal microorganisms must be cultured from two or more blood cultures taken on separate occasions, no more than one day apart [18,19];
- Catheter-Associated Bloodstream Infection 3: For children under 1 year, three additional signs and symptoms are added based on Definition 2 (not applicable to this study) [18,19].

### 2.3. Inclusion and Exclusion Criteria

Inclusion:

- Individuals aged 18 years and older;
- Hospitalization longer than 48 h after admission;
- Admission without a catheter;
- Admission without antimicrobial treatment;
- Not hospitalized in another institution in the previous month;
- Accessibility to digital medical records.

Exclusion:

- Presence of prior infection, whether related or unrelated to the catheter;
- Patients with bacteremia originating from a source other than the central vascular catheter.

### 2.4. Analysis Plan

To determine whether each variable followed a normal distribution, the Shapiro–Wilk test was employed, assuming the null hypothesis that the data are normally distributed. Variables were matched based on hospitalization date, age, and Charlson score to control for confounding factors. Significant differences between continuous variables were assessed using the Student’s *t*-test for normally distributed data and the Wilcoxon Mann–Whitney test for non-normal distributions. Categorical variables were analyzed using either the chi-square test or Fisher’s exact test, depending on the number of observations.

The median duration of catheter use, hospitalization, and time with the catheter in place were measured for both CLABSI-exposed and non-exposed patients. Bivariate analysis was conducted to evaluate the relationship between each independent variable and CLABSI exposure, characterizing the cohort both clinically and demographically. In the case–control analysis, patients were also characterized based on their discharge status. A conditional logistic regression model was used to estimate the odds of death for patients with CLABSI, adjusting for age, sex, Charlson index (categorized into two groups: 0–3 and 4+), type of hospitalization, previous hospitalizations, and time from catheter placement to discharge (categorized into percentiles: 25, 50, 75, and above 75). The model further categorized the CLABSI variable into three levels: no bacteremia, CLABSI without resistance, and CLABSI with resistance. The significance level for all tests was set at 0.05. For patients with bacteremia, the frequencies of each etiological agent were calculated. Logistic regression was used to estimate the odds of death for patients with resistant infections, adjusted for age, sex, Charlson index, type of hospitalization, previous hospitalizations, and time from catheter placement to discharge. All analyses were conducted using STATA 14^®^ (accessed on 6 April 2023, https://www.stata.com).

### 2.5. Ethics Considerations

This observational, retrospective study collected data from reports on adult patients and their clinical histories, excluding vulnerable populations. Conducted in alignment with the Helsinki Declaration, the study aimed to promote health and protect patient rights, ensuring confidentiality, dignity, integrity, and privacy. Data were gathered exclusively by the master’s student in epidemiology, aided by a technically trained research assistant. Both were appropriately educated and trained, and the study received approval from the institutional research and ethics committees (Act No. IYECDO-0804, 23 November 2018; Act No. IYECDO-0806, 27 November 2018) and the Institutional Committee of Human Ethics (CIREH) at Universidad del Valle (Act No. 020-019, 29 November 2019).

## 3. Results

### 3.1. Patient Flowchart

In this study, the occurrence and impact of central line-associated bloodstream infection (CLABSI) in a hospital setting were analyzed. The selection of patients was conducted meticulously to ensure reliability and validity.

**Figure 1:**
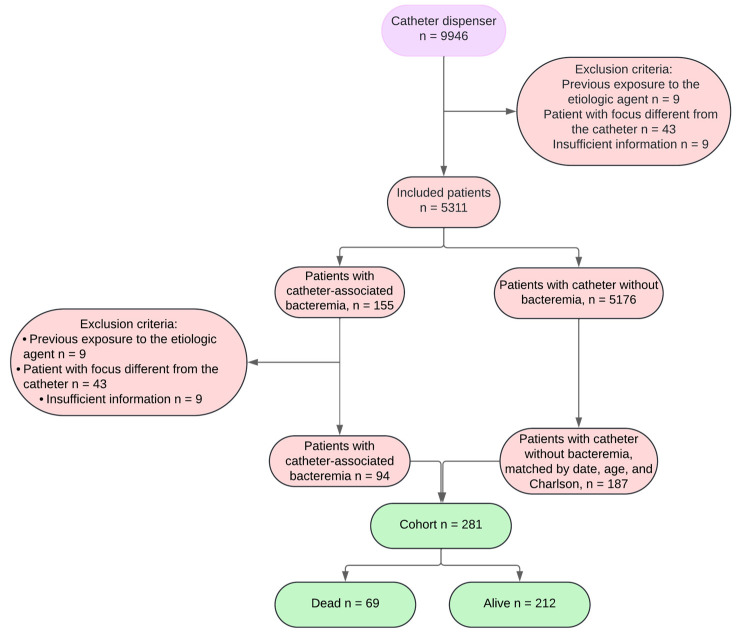
Patient selection flow diagram.

As illustrated in Figure 1, the data collection process identified 9946 dispensations. From these, 4615 dispensations were excluded: 404 due to patients being under 18 years old and 4211 due to repeated dispensations for the same patient. This exclusion process resulted in a cohort of 5311 unique patients who had received CL.

From the 5311 patients, 155 cases with documented CLABSI, as recorded by the institutional infection control committee, were extracted. The remaining 5176 patients had CL without infection. Further exclusions within the CLABSI group included 9 patients with prior infections, 43 patients with bacteremia from sources other than the catheter, and 9 cases with insufficient information regarding catheter placement (due to placement in another institution). This refined the CLABSI group to 94 patients.

These 94 CLABSI patients were then matched with two controls each, based on age, Charlson index, and catheter placement/CLABSI date, drawn from the 5176 non-infected CL patients. This matching process resulted in a control group of 187 patients who did not have CLABSI (in some instances, only one control was found per CLABSI patient). Consequently, a cohort of 281 patients with CL was established.

The outcomes at the end of the observation period identified 69 patients who had died and 212 who were alive. This detailed selection and matching process ensured a robust comparative analysis of the CLABSI impact on patient outcomes.

### 3.2. Clinical and Demographic Characteristics of Patients with and without Catheter-Associated Bacteremia

Table 1 presents the demographic and clinical characteristics of patients with and without catheter-associated bacteremia. There were no significant differences in sex distribution (*p* = 0.764). The median age of the population was 63 years (IQR 48–75), with no significant differences between the groups (*p* = 0.862). Notably, 42 patients (44.7%) in the bacteremia group were in the ICU at the time of diagnosis, compared to 55 patients (29.4%) in the non-bacteremia group, indicating a significant difference (*p* = 0.011).

Further analysis revealed that, in the bacteremia group, the prevalence of dementia (*p* < 0.001), chronic respiratory disease (*p* = 0.001), moderate/severe chronic renal insufficiency (*p* = 0.024), and cancer of any type (*p* = 0.003) was significantly higher compared to the non-bacteremia group. Additionally, the site of catheter placement differed between the groups, with femoral insertion being more common in patients who developed CLABSI. Furthermore, a history of previous hospitalizations and prior antibiotic administration before catheter insertion were more frequent in the bacteremia group (*p* < 0.001). However, the Charlson score at admission did not differ significantly between the groups (*p* = 0.823).

Regarding time-related variables, the group exposed to CLABSI exhibited significantly longer durations in all measured aspects: hospital stay duration (median = 51 vs. median = 17, *p* = 0.000), catheter duration (portability) (median = 7 vs. median = 19, *p* < 0.001), and time from catheter insertion to discharge (median = 40 vs. median = 9, *p* < 0.001).

**Figure 2:**
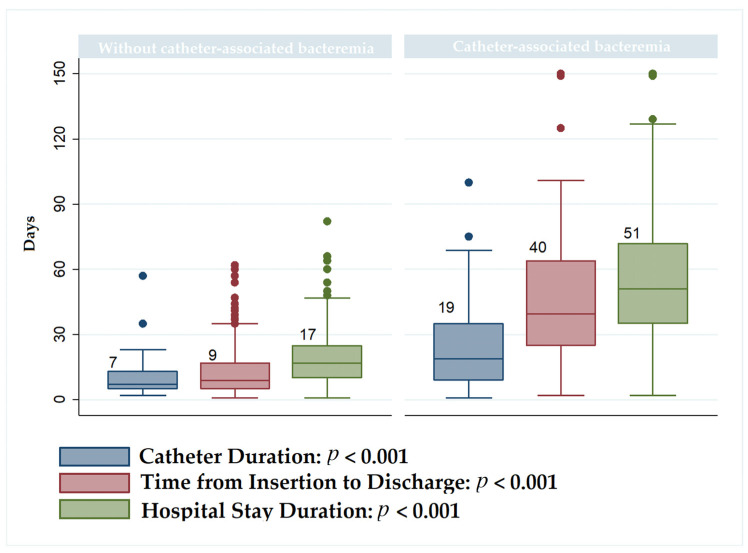
Comparison of catheter duration, insertion to discharge time, and hospital stay duration between groups with and without bacteremia.

Figure 2 illustrates the behavior of catheter duration (portability), the time from catheter insertion to discharge, and the duration of hospital stay for both the cohort exposed to catheter-associated bacteremia and the cohort not exposed to it.

### 3.3. Clinical and Demographic Characteristics of Patients According to Discharge Status

Table 2 provides a detailed comparison of the demographic and clinical characteristics of living and deceased patients for the case–control analysis. The analysis revealed that a higher percentage of deceased patients were women (65.2%, *p* = 0.039). Age also differed significantly between the two groups, with deceased patients being older on average (*p* = 0.033). Additionally, 48 patients (69.6%) in the deceased group were in the ICU at the time of death, compared to 49 patients (23.1%) in the living group, a difference that was statistically significant (*p* = 0.000).

In terms of comorbidities, the most common among deceased patients were heart failure, acute myocardial infarction, moderate to severe chronic renal insufficiency, and cancer of any type. However, no significant differences were observed when compared to the living group. The Charlson score did not differ significantly between the groups (*p* = 0.640). The deceased group had a higher percentage of previous hospitalizations compared to the control group (*p* = 0.038). Furthermore, the anatomical site of catheter placement varied between the groups (*p* = 0.048), with subclavian insertion being more common among deceased patients. Interestingly, the control group required more surgeries during hospitalization than the deceased group (*p* = 0.08).

**Figure 3:**
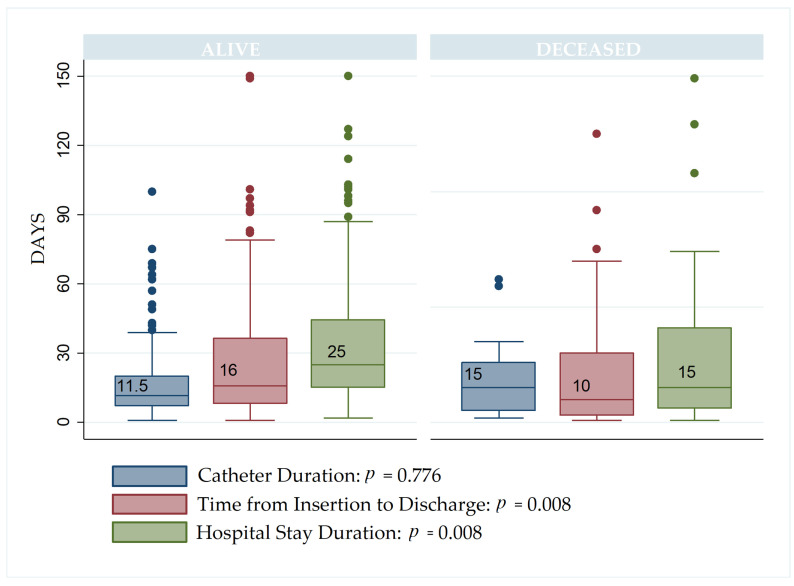
Comparison of catheter duration, insertion to discharge time, and hospital stay between alive and deceased patients.

Figure 3 illustrates the comparison of catheter duration, time from insertion to discharge, and hospital stay duration between living and deceased patients. The median catheter duration was 11.5 days for living patients and 15 days for deceased patients (*p* = 0.776). The time from catheter insertion to discharge showed a significant difference, with medians of 16 days for living patients and 10 days for deceased patients (*p* = 0.008). Additionally, hospital stay duration was significantly longer for living patients (median of 25 days) compared to deceased patients (median of 15 days) (*p* = 0.008).

### 3.4. Relationship between Individual Characteristics and Patient Mortality

Table 3 describes the univariate and multivariate analysis for mortality using a logistic model conditioned by hospitalization date, conducted with 249 patients (69 cases matched to 180 controls). In the univariate logistic regression analysis, it was found that a patient with catheter-associated bacteremia has 1.80 times the odds of dying compared to a patient without it, but this association was not significant (95% CI [0.95–3.39], *p* = 0.069). However, in the multivariate analysis adjusting for sex, age, type of hospitalization, previous hospitalizations, time from catheter placement to discharge, and categorized Charlson score, it was found that a patient with catheter-associated bacteremia has 3.89 times the odds of dying compared to a patient without it, and this association was statistically significant (95% CI [1.33–11.31], *p* = 0.013). For each additional year of age, the odds of dying increase by 1.07 times (95% CI [1.01–1.13], *p* = 0.021). Being hospitalized in the ICU increases the odds of dying by 6.98 times (95% CI [3.10–15.73], *p* = 0.000) compared to those not in the ICU. For each day that passes since catheterization, the risk of dying decreases by 3% (*p* = 0.022). Patients with a catheter placement duration of 0 to 6 days have 8.40 times the odds of dying compared to those with a duration of 35 days or more (reference category—Appendix A). Moderate/severe renal disease was not considered in the multivariate analysis as it is included in the Charlson score evaluation.

Table 4 shows that categorizing the CLABSI variable into three levels (no bacteremia, CLABSI without resistance, and CLABSI with resistance) changes some estimates in the multivariate model without affecting the directionality of any variable. The odds of dying for patients who develop CLABSI without resistance is 2.3 times compared to patients without CLABSI (95% CI [0.69–7.71], *p* = 0.177). The odds of dying for patients who develop CLABSI with resistance is 6.98 times compared to patients without CLABSI (95% CI [1.92–25.97], *p* = 0.003). The odds of dying for men is 65% lower than for women (95% CI [0.15–0.79], *p* = 0.011). Being hospitalized in the ICU increases the odds of dying by 7.22 times compared to those not in the ICU (95% CI [3.16–16.50], *p* = 0.000).

According to the Wald test (*p* = 0.0001), the variables catheter-associated bacteremia, sex, age, Charlson score, type of hospitalization, previous hospitalizations, and time from catheter placement to discharge form a useful model to explain the discharge status of a patient. No interaction was found between the variable catheter-associated bacteremia and the other variables included in the model.

### 3.5. Description of Isolated Germs

Table 5 outlines the frequency of pathogens in patients with bacteremia based on their discharge status. The most frequently isolated pathogen was *Klebsiella pneumoniae*, found in 19 patients (20.2%), followed by *Staphylococcus aureus*, identified in 14 patients (14.9%). Among the patients infected with *Klebsiella pneumoniae*, 5 (26.3%) died. In contrast, 6 (42.8%) of the patients with *Staphylococcus aureus* infection died. Additionally, *Stenotrophomonas maltophilia* was isolated in three patients, with two of these patients succumbing to the infection.

### 3.6. Relationship between Individual Characteristics and Mortality of Patients with Catheter-Associated Bacteremia

Table 6 presents the univariate and multivariate analysis of discharge status in patients with CLABSI according to the resistance report. In the univariate analysis, it was observed that patients with bacteremia caused by resistant microorganisms have 2.62 times the odds of dying compared to those with bacteremia caused by non-resistant organisms. However, this association was not statistically significant (95% CI [0.96–7.04], *p* = 0.104).

In the multivariate analysis, after adjusting for variables such as sex, age, categorized Charlson score, time from catheter insertion to discharge, and previous hospitalizations, it was found that patients with resistant microorganisms have 4.04 times the odds of dying compared to those with bacteremia caused by non-resistant organisms. This association was statistically significant (95% CI [1.17–13.96], *p* = 0.018).

The Pearson goodness-of-fit test yielded a p-value of 0.5353, which does not allow us to reject the null hypothesis and confirms that the model’s estimates fit the theoretical model well. No interaction was found between the variable of catheter-associated bacteremia and the other variables included in the model.

## 4. Discussion

In a tertiary and quaternary healthcare institution in Cali, CLABSI has been identified as a significant mortality risk factor for hospitalized patients. This was confirmed through an adjusted conditional logistic model, showing that patients with CLABSI have a 3.89 times higher risk of death (95% CI [1.33–11.31], *p* = 0.013) compared to those without this infection. Studies have shown that patients with CLABSIs have a 2.27-fold increased risk of mortality (Stevens et al., 2014) [20] and are 36.6% more likely to die in the hospital (Chovanec et al., 2021) [21].

While our findings indicate a strong association between CLABSI and increased mortality risk, it is important to clarify that this study does not definitively establish causality. The observed association may be influenced by the fact that CLABSI often occurs in patients who are already critically ill, which could contribute to the higher mortality rates in this group. For instance, Mishra et al., 2016 [22] found that patients with higher SOFA and APACHE II scores, both indicators of severe illness, were more likely to develop CLABSI and subsequently had higher mortality rates. Their study suggested that CLABSI might serve more as a marker of critical illness rather than a direct cause of mortality. Similarly, Alwazzeh et al., 2023 [23] reported a strong association between CLABSI and increased mortality, particularly among critically ill patients with multiple comorbidities, emphasizing the challenges in determining causality due to the patients’ complex health conditions.

Further research is needed to determine whether CLABSI directly contributes to mortality or primarily reflects underlying severe illness. Distinguishing between association and causality is crucial for accurately assessing the impact of CLABSI on patient outcomes and for developing more targeted interventions.

This finding aligns with global studies, emphasizing the need to strengthen healthcare-associated infection prevention protocols, particularly for CLABSI [7,20,24]. Bell and O’Grady highlight multiple strategies for preventing CLABSI, including education, antiseptic use, barrier precautions, and advanced technologies such as antibiotic-impregnated catheters and sutureless devices [25,26,27].

Bacterial resistance significantly impacts patient outcomes, with multivariate logistic analysis showing that patients with resistant infections have a 4.04 times higher mortality risk (95% CI [1.17–13.96], *p* = 0.027). For non-resistant CLABSI, the mortality risk was 2.3 times higher (95% CI [0.69–7.71], *p* = 0.177), while resistant CLABSI showed a 6.98 times higher risk (95% CI [1.92–25.97], *p* = 0.003). The broader confidence interval suggests that the sample size may not have been sufficient to determine the exact association. *Staphylococcus aureus*, the second most common pathogen (n = 14) after *Klebsiella pneumoniae* (n = 19), showed a high mortality rate, with 92.8% being methicillin-resistant. These findings underscore the need for stringent protocols to control bacterial resistance [24,28,29,30,31].

Patients with bacteremia had significantly longer hospital stays (median = 51 days vs. 17 days; *p* = 0.000) and catheter duration (median = 19 days vs. 7 days; *p* < 0.001) than those without bacteremia. When considering hospitalization time from catheter insertion to discharge or death, the median was also higher for the bacteremia group (median = 40 days vs. 9 days; *p* < 0.001). These results are consistent with other studies showing longer hospital stays for patients with CLABSI [24,29].

In the conditional logistic model, ICU patients had a 6.98 times higher mortality risk (95% CI [3.1–15.733], *p* = 0.000) compared to non-ICU patients, indicating a significant association between ICU admission and mortality due to the critical nature of their conditions. A 2016 Colombian study reported a 32% mortality rate in ICU patients, with higher severity scores (APACHE II) among those who died (median = 28 vs. 18; *p* < 0.05). Cardiovascular and neurological admissions had higher mortality risks (OR: 5.0, *p* = 0.0564; OR: 5.55, *p* = 0.022) [32].

Previous hospitalization increased the mortality risk by 45% (OR = 1.45; 95% CI [0.6–3.48], *p* = 0.407); although not statistically significant, it suggests a positive trend. The conditional logistic model for catheter-to-discharge time showed an 8.40 times higher mortality risk within the first six days post-catheterization compared to 35 days or more (95% CI [2.1–33.61], *p* = 0.003), indicating that early complications from catheter insertion significantly increase mortality risk [33,34].

Patients with underlying conditions like diabetes (OR = 3.0, *p* = 0.039), COPD (OR = 3.3, *p* = 0.078), and metastatic cancer (OR = 2.3, *p* = 0.083) had higher complication risks associated with catheterization [35,36] (Aw et al., 2012; Gao et al., 2020). Ultrasonography-guided catheterization reduces mechanical complications and the number of attempts needed for successful cannulation [25].

Huerta et al., 2018 [37] conducted a retrospective cohort study that identified several key factors associated with recurrence and mortality in hospital-acquired central line-associated bloodstream infections. They found that shorter antimicrobial treatment durations were significantly associated with increased mortality and recurrence rates within 60 days post-treatment. Specifically, the hazard ratio for mortality or recurrence with shorter treatment duration was 0.35 (95% CI 0.26–0.48), indicating a protective effect of prolonged antimicrobial therapy. Additionally, higher SOFA scores and increased age were also associated with worse outcomes. The study suggests that while longer antimicrobial treatments beyond 15 days might not significantly improve outcomes further, they are critical in reducing early mortality and recurrence. These findings emphasize the importance of optimizing antimicrobial therapy duration and considering patient-specific factors like age and severity of illness when managing CLABSI. 

In the study by Satta et al., 2023 [38], the researchers highlighted the significant increase in CLABSI rates during the COVID-19 pandemic, attributing this rise to healthcare system pressures, such as high staff workloads and redeployment. They found that lapses in infection control practices, such as hand hygiene and adherence to line care bundles, likely contributed to this increase. The study underscores the need for strict adherence to infection control measures, especially during periods of increased healthcare strain. Additionally, the use of advanced technologies like antibiotic-impregnated catheters and continuous staff education were recommended as key strategies to mitigate CLABSI risk.

Maqbool et al., 2023 [39] conducted a prospective study to determine the incidence of CLABSI in a tertiary care hospital in Northern India. They found that the incidence of CLABSI was 9.3 per 1000 catheter days and 6.7 per 1000 inpatient days, with a device utilization ratio of 0.7. The most common pathogens isolated were Acinetobacter species (22%), followed by *Klebsiella pneumoniae* (16%) and *Enterobacter aerogenes* (16%). The study highlighted that Gram-negative organisms were predominant (72%) and displayed high resistance to multiple antibiotics. For instance, polymyxin B showed the highest sensitivity among Gram-negative organisms (100%). Among Gram-positive organisms, *Staphylococcus aureus* exhibited 100% sensitivity to vancomycin, teicoplanin, and linezolid. The mortality rate for patients with CLABSI was notably higher (62.2%) compared to those without bloodstream infections (58.4%), underscoring the severe impact of CLABSI on patient outcomes.

Leblebicioglu et al., 2013 [40] conducted a study on the impact of a multidimensional infection control approach on CLABSI rates in adult intensive care units across eight cities in Turkey. They implemented a comprehensive infection control strategy that included a bundle of interventions, education, outcome surveillance, process surveillance, and feedback on CLABSI rates and performance. The study revealed a significant reduction in CLABSI rates, from 22.7 per 1000 central line days during the baseline period to 12.0 per 1000 central line days during the intervention period. This amounted to a 39% reduction in CLABSI incidence, demonstrating the effectiveness of a multidimensional approach in reducing infection rates.

Baier et al., 2020 [41] conducted a retrospective cohort study on the incidence, risk factors, and healthcare costs associated with CLABSI in hematologic and oncologic patients. The study found a CLABSI incidence rate of 10.6 cases per 1000 CL days and identified several independent risk factors for CLABSI, including the use of more than one CL per case, CL insertion for conditioning, acute myeloid leukemia, leukocytopenia, carbapenem therapy, and pre-existing pulmonary diseases. Interestingly, the study also identified protective factors such as erythrocyte transfusions, glycopeptide therapy, and the use of the subclavian vein as the CL insertion site. The study revealed that CLABSI significantly increased hospital costs, with attributable median costs of EUR 8810 per case. These costs resulted in a median loss of EUR 8171 per CLABSI case, as costs exceeded reimbursements. This underscores the significant economic burden of CLABSI on healthcare systems.

Larsen et al., 2019 [42] conducted a systematic review to evaluate the reliability and accuracy of CLABSI diagnostic reporting using the National Health and Safety Network (NHSN) criteria across various hospitals internationally. The review found that publicly reported CLABSI rates were frequently underestimated, with sensitivity ranging from 0.42 to 0.88 and specificity ranging from 0.70 to 0.99. The study highlighted that this underestimation of true CLABSI incidence could significantly impact hospital benchmarking, quality improvement initiatives, and the allocation of resources. Moreover, the variability in applying the NHSN/CDC criteria across different institutions led to inconsistencies in CLABSI reporting, potentially leading to underreporting. These discrepancies underscore the importance of accurate surveillance and the need for regular audits, education, and proper resource allocation to improve the reliability of CLABSI data reporting.

Mishra et al., 2017 [22] conducted a prospective observational study to evaluate the incidence, risk factors, and associated mortality of CLABSI in an adult intensive care unit (ICU) in Northern India. The study revealed that the overall rate of CLABSI was 17.04 per 1000 catheter days and 14.21 per 1000 inpatient days, significantly higher than rates reported in developed countries. *Klebsiella pneumoniae* was the most common pathogen identified, accounting for 40% of the cases, and the study highlighted the high prevalence of multidrug-resistant organisms. The study identified several significant risk factors for developing CLABSI, including immunosuppression, age over 60 years, duration of central line placement exceeding 10 days, and ICU stay longer than 21 days. The mortality rate associated with CLABSI was high, at 56%, with immunosuppression and extended central line duration being independent predictors of acquiring CLABSI.

Alwazzeh et al., 2023 [23] conducted a retrospective observational study to investigate the microbiological trends and mortality risk factors associated with CLABSI in a tertiary medical center in Saudi Arabia between 2015 and 2020. The study reported a 33.6% overall 30-day mortality rate among 214 patients diagnosed with CLABSI. The study highlighted an alarming increase in the prevalence of Candida spp. infections, rising from 13% in 2015 to 24% in 2020, with a predominance of Gram-negative pathogens. Multidrug-resistant organisms (MDROs) were found in 47% of bacterial CLABSI cases, underscoring the significant challenge of antibiotic resistance in the management of these infections. The study also identified several comorbidities that were significantly associated with higher mortality in CLABSI patients, including diabetes mellitus, cardiovascular diseases, lung diseases, chronic kidney disease, and having three or more comorbidities. These findings suggest that patients with multiple comorbidities are at a particularly high risk of poor outcomes when they develop CLABSI.

Comparative data from other studies reinforce the significance of these findings. For instance, a study from an academic medical center reported that the overall 30-day mortality rate for CLABSI patients was 33.6%, with significant mortality differences based on central line type and insertion site (*p* = 0.0478 and *p* = 0.0034, respectively) [43]. Another study highlighted that shorter antimicrobial treatment duration, higher SOFA scores, increasing age, and the presence of cirrhosis or femoral central lines were associated with increased 60-day mortality or recurrence [37,44,45]. These additional data points underscore the multifactorial nature of CLABSI-related mortality and the importance of comprehensive infection control measures. Huerta et al. (2018) conducted a retrospective cohort study that identified significant factors associated with recurrence and mortality in hospital-acquired central line-associated bloodstream infections (HA-CLABSIs). They found that shorter antimicrobial treatment durations were linked to higher mortality and recurrence rates within 60 days post-treatment, with a hazard ratio of 0.35 (95% CI 0.26–0.48). Additionally, higher SOFA scores and advanced age were also associated with worse outcomes. These findings align with our study, which also identified age and the severity of illness as critical factors influencing patient outcomes. However, our study emphasizes the importance of ICU admission as a major risk factor, with ICU patients showing a 6.98 times higher mortality risk.

Our findings indicate an association between CLABSI and increased mortality, particularly in patients with comorbidities, advanced age, and those in the ICU. However, this study does not definitively establish causality. The predominance of Gram-negative organisms and the heightened risk associated with resistant strains highlight the urgent need for targeted antimicrobial stewardship and infection control protocols. Prolonged hospital stays and catheter durations exacerbate outcomes and increase healthcare costs. Addressing these issues requires strict enforcement of infection control practices, optimizing antibiotic use, reducing unnecessary catheter days, and investing in advanced technologies like antibiotic-impregnated catheters. Additionally, ongoing staff education and training on CLABSI prevention are crucial to improving patient outcomes and reducing infection incidence. Developing more refined protocols and targeted interventions that consider these complexities is essential for enhancing patient care and minimizing the impact of CLABSI.

## 5. Limitations and Strengths

This study faced several limitations, primarily due to the retrospective nature of data collection, which made it challenging to eliminate misclassification bias. Specifically, we were unable to reconstruct the APACHE II severity score for many patients, as the necessary examinations were not consistently performed during their hospital stays. Additionally, follow-up data were incomplete after patient discharge, leaving a gap in understanding long-term survival outcomes.

Furthermore, the study did not include comparative data or catheter days infection rates, which are crucial metrics for improving protocols and assessing the effectiveness of infection control measures. The absence of systematic and comparative records of catheter days in Colombian hospitals, potentially due to limitations in data infrastructure and the implementation of international practices, may have hindered a comprehensive analysis of CLABSI rates. While various guidelines, such as the Clinical Practice Guidelines for the Prevention of Nosocomial Infections Associated with Medical Devices (Álvarez et al., 2010 [46]), the Protocol for Public Health Surveillance (Martínez et al., 2016 [47]), and the Manual of Basic Measures for Infection Control in Healthcare Facilities (Gaviria et al., 2018 [48]), emphasize infection control, their implementation and adherence across institutions might not be consistent. This possible inconsistency, coupled with the lack of standardized data collection protocols as recommended by organizations like the CDC, WHO, and IDSA, further limited the scope of our findings.

However, the study had significant strengths. The institutional information systems allowed for easy access to complete medical records and reliable microbiological and laboratory reports, ensuring robust and consistent data. The significant associations observed were both statistically and theoretically sound, enhancing the validity of the results.

For future studies, a prospective design is recommended to capture more comprehensive and timely data, including APACHE II scores and catheter days infection rates. Implementing standardized protocols for data collection and ensuring regular follow-up visits can provide a more accurate picture of long-term patient outcomes. Strengthening the integration of electronic health records across institutions will also improve data accessibility and research quality.

## 6. Conclusions

This study highlights a strong association between CLABSI and poorer patient outcomes, including extended hospital stays and increased mortality, particularly in critically ill patients. However, it is important to clarify that this study does not confirm a direct causal link between CLABSI and mortality. The impact of bacterial resistance further exacerbates these risks, underscoring the need for improved infection control measures and judicious antibiotic use. Enhancing these practices, along with investing in advanced technologies and ongoing staff education, is essential to reduce the incidence and severity of CLABSI.

## Figures and Tables

**Table 1:** Clinical and sociodemographic description of the cohort.

Characteristics	Without Bacteremia (n = 187)	With Bacteremia (n = 94)	*p*-Value	Test Used
**Sex, n (%)**				
Female	103 (55.1)	50 (53.2)	0.764	Chi-squared test
Male	84 (44.9)	44 (46.8)		
**Age [years], median (IQR)**	64 (49–75)	63 (48–74)	0.862	Mann–Whitney
**Social security regimen, n (%)**				
Contributive	163 (87.2)	87 (92.6)	0.174	Chi-squared test
Subsidized	24 (12.8)	7 (7.4)		
**Type of hospitalization, n (%)**				
ICU	55 (29.4)	42 (44.7)	0.011	Chi-squared test
Non-ICU	132 (70.6)	52 (55.3)		
**Comorbidities, n (%)**				
Ischemic disease	56 (29.9)	22 (23.4)	0.248	Chi-squared test
Heart failure	64 (34.2)	37 (39.4)	0.397	Chi-squared test
Dementia	0 (0)	10 (10.6)	<0.001	Fisher’s exact test
Chronic respiratory disease	12 (6.4)	18 (19.1)	0.001	Chi-squared test
Connective tissue disease	26 (13.9)	15 (16.0)	0.645	Chi-squared test
Diabetes	51 (27.3)	20 (21.3)	0.275	Chi-squared test
Hemiplegia	14 (7.5)	4 (4.3)	0.219	Fisher’s exact test
Moderate/severe chronic renal insufficiency	42 (22.5)	33 (35.1)	0.024	Chi-squared test
Hepatic disease	8 (4.3)	3 (3.2)	0.658	Fisher’s exact test
Cancer	59 (31.6)	42 (44.7)	0.030	Chi-squared test
Defined AIDS	2 (1.1)	1 (1.1)		
**Previous hospitalizations**	113 (60.4)	83 (88.3)	<0.001	Chi-squared test
**Previous antibiotics during hospitalization**	114 (61.0)	94 (100.0)	<0.001	Fisher’s exact test
**Charlson score**				
0 to 1	12 (6.4)	5 (5.3)	0.823	Fisher’s exact test
2 to 3	30 (16.0)	17 (18.1)		
4 or more	145 (77.5)	72 (76.6)		
**Catheter insertion site, n (%)**				
Jugular	46 (24.6)	16 (17.0)	0.019	Fisher’s exact test
Subclavian	58 (31.0)	26 (27.6)		
Femoral	10 (5.3)	12 (12.8)		
Superior vena cava	0 (0)	3 (3.2)		
Other	73 (39.0)	37 (39.4)		
**Catheter duration [days], median (IQR)**	7 (5–13)	19 (9–35)	<0.001	Mann–Whitney
**Surgery**	123 (65.8)	68 (72.3)	0.266	Chi-squared test
**Time from catheter insertion to discharge**	9 (5–17)	40 (25–64)	<0.001	Mann–Whitney
**Hospital stay duration, median (IQR)**	17 (10–25)	51 (35–72)	<0.001	Mann–Whitney
**Discharge status, n (%)**				
Alive	146 (78.1)	66 (70.2)	0.149	Chi-squared test
Deceased	41 (21.9)	28 (29.8)		

**Table 2:** Clinical and demographic characterization of hospitalized patients with central vascular catheter according to discharge status.

Characteristic	Alive (n = 212)	Deceased (n = 69)	*p*-Value
**Sex**			
Female	108 (50.9)	45 (65.2)	0.04
Male	104 (49.1)	24 (34.8)	
**Age [years], median (IQR)**	62 (36–73)	68 (48–79)	0.033
**Social security regimen**			
Subsidized	187 (88.2)	63 (91.3)	0.48
Contributive	25 (11.8)	6 (8.7)	
**Type of hospitalization**			
Non-ICU	163 (76.9)	21 (30.4)	0.000
ICU	49 (23.1)	48 (69.6)	
**Comorbidities**			
Ischemic disease	55 (25.9)	23 (33.3)	0.954
Heart failure	76 (35.8)	25 (36.2)	0.248
Dementia	6 (2.8)	4 (5.8)	
Chronic respiratory disease	25 (11.8)	5 (7.2)	0.288
Connective tissue disease	33 (15.6)	8 (11.6)	0.417
Mild chronic liver disease	9 (4.2)	2 (2.9)	
Diabetes	5 (2.4)	1 (1.4)	
Hemiplegia	6 (2.8)	1 (1.4)	
Moderate/severe chronic renal insufficiency	48 (22.6)	27 (39.1)	0.007
Cancer	13 (6.1)	9 (13.0)	0.561
Previous hospitalizations	141 (66.5)	55 (79.7)	0.038
Previous antibiotics during hospitalization	152 (71.7)	56 (81.2)	0.120
**Catheter insertion site**			
Jugular	46 (21.7)	16 (23.2)	0.05
Subclavian	57 (26.9)	27 (39.1)	
Femoral	16 (7.5)	6 (8.7)	
Superior vena cava	1 (0.5)	2 (2.9)	
Other	92 (43.4)	18 (26.1)	
**Charlson score**			
0 to 1	14 (6.6)	3 (4.3)	0.64
2 to 3	37 (17.5)	10 (14.5)	
4 or more	161 (75.9)	56 (81.2)	
**Catheter duration [days], median (IQR)**	11.5 (7–20)	15 (5–26)	0.776
**Surgery**	150 (70.8)	41 (59.4)	0.08
**Time from catheter insertion to discharge**	16 (8–36.5)	10 (3–30)	0.008
**Hospital stay duration, median (IQR)**	25 (15–44.5)	15 (6–41)	0.008
**Catheter-associated bacteremia**	66 (31.1)	28 (40.6)	0.149

**Table 3:** Relationship between individual characteristics and patient mortality, 253 patients.

Variables	Univariate Analysis				Multivariate Analysis			
	OR	EE	IC95%	*p*-Value	OR	EE	IC95%	*p*-Value
Sex (female = 0, male = 1)	0.51	0.15	0.28–0.92	0.025	0.44	0.16	0.21–0.91	0.028
Age (18 or older)	1.06	0.02	1.02–1.11	0.004	1.07	0.03	1.01–1.13	0.021
Charlson category (score 0 to 3 = 1, 4 or more = 1)	4.73	3.32	1.19–18.76	0.027	2.41	2.19	0.40–14.33	0.335
CLABSI (no = 0, yes = 1)	1.80	0.58	0.95–3.39	0.069	3.89	2.12	1.33–11.31	0.013
Type of hospitalization (non-ICU = 0, ICU = 1)	7.52	2.72	3.70–15.29	0.000	6.98	2.89	3.10–15.73	0.000
Previous hospitalizations (no = 0, yes = 1)	2.14	0.74	1.09–4.22	0.027	1.45	0.65	0.60–3.48	0.407
Time from catheter to discharge (2 or more days)	0.99	0.01	0.98–1.00	0.134	0.97	0.01	0.95–1.00	0.022

**Table 4:** Relationship between individual characteristics and patient mortality (CLABSI categorized), 253 patients.

Variables	Univariate Analysis				Multivariate Analysis			
	OR	EE	IC95%	*p*-Value	OR	EE	IC95%	*p*-Value
**Sex (female = 0, male = 1)**	0.51	0.15	0.28–0.92	0.025	0.35	0.14	0.15–0.79	0.011
**Age (18 or older)**	1.06	0.02	1.02–1.11	0.004	1.07	0.03	1.01–1.13	0.018
**Charlson category (score 0 to 3 = 1, 4 or more = 1)**	4.73	3.32	1.19–18.76	0.027	2.56	2.38	0.41–15.89	0.313
**CLABSI (reference category = no bacteremia)**								
CLABSI/without resistance	1.42	0.578	0.64–3.15	0.39	2.3	1.42	0.69–7.71	0.177
CLABSI/with resistance	2.23	0.854	1.05–4.72	0.037	6.96	4.58	1.92–25.27	0.003
**Type of hospitalization (non-ICU = 0, ICU = 1)**	7.52	2.72	3.70–15.29	0	7.22	3.04	3.16–16.50	0
**Previous hospitalizations (no = 0, yes = 1)**	2.14	0.74	1.09–4.22	0.027	1.43	0.65	0.59–3.47	0.427
**Time from catheter to discharge (2 or more days)**	0.99	0.01	0.98–1.00	0.134	0.98	0.01	0.96–0.99	0.026

**Table 5:** Isolated pathogens in patients with bacteremia according to discharge status.

Isolated Pathogen	Alive, n = 66	Deceased, n = 28	Total, n = 94
**Gram-negative, n (%)**			
*Acinetobacter lwoffii*	1 (1.52)	0 (0)	1 (1.06)
*Acinetobacter baumannii*	3 (4.55)	0 (0)	3 (3.19)
*Burkholderia (P.) cepacia*	0 (0)	1 (3.57)	1 (1.06)
*Chryseobacterium (F.)*	1 (1.52)	0 (0)	1 (1.06)
*Enterobacter aerogenes*	0 (0)	1 (3.57)	1 (1.06)
*Enterobacter cloacae*	1 (1.52)	0 (0)	1 (1.06)
*Escherichia coli*	7 (10.61)	2 (7.14)	9 (9.57)
*Klebsiella oxytoca*	0 (0)	1 (3.57)	1 (1.06)
*Klebsiella pneumoniae*	14 (21.21)	5 (17.86)	19 (20.21)
*Morganella morganii*	1 (1.52)	0 (0)	1 (1.06)
*Pseudomonas aeruginosa*	5 (7.58)	2 (7.14)	7 (7.45)
*Pseudomonas species*	1 (1.52)	0 (0)	1 (1.06)
*Pseudomonas fluorescens*	1 (1.52)	0 (0)	1 (1.06)
*Serratia marcescens*	8 (12.12)	1 (3.57)	9 (9.57)
*Stenotrophomonas maltophilia*	1 (1.52)	2 (7.14)	3 (3.19)
**Gram-positive, n (%)**			
*Staphylococcus aureus*	8 (12.12)	6 (21.43)	14 (14.89)
*Staphylococcus capitis*	1 (1.52)	0 (0)	1 (1.06)
*Staphylococcus epidermidis*	1 (1.52)	2 (7.14)	3 (3.19)
*Staphylococcus sciuri*	2 (3.03)	0 (0)	2 (2.13)
*Enterococcus faecalis*	2 (3.03)	0 (0)	2 (2.13)
*Enterococcus faecium*	0 (0)	1 (3.57)	1 (1.06)
**Yeasts, n (%)**			
*Candida albicans*	2 (3.03)	1 (3.57)	3 (3.19)
*Candida famata*	2 (3.03)	1 (3.57)	3 (3.19)
*Candida glabrata*	1 (1.52)	0 (0)	1 (1.06)
*Candida parapsilosis*	1 (1.52)	1 (3.57)	2 (2.13)
*Candida tropicalis*	0 (0)	1 (3.57)	1 (1.06)

**Table 6:** Relationship between individual characteristics and mortality in patients with CLABSI, total n = 84.

Variables	Univariate Analysis				Multivariate Analysis			
	OR	EE	IC95%	*p*-Value	OR	EE	IC95%	*p*-Value
Sex (female = 0, male = 1)	0.65	0.297	0.26–1.59	0.343	1	0.02	0.96–1.05	0.923
Age (18 or older)	1.01	0.014	0.98–1.04	0.43	0.32	0.21	0.09–1.17	0.085
Charlson category (score 0 to 3 = 1, 4 or more = 1)	1.17	0.637	0.4–3.4	0.768	1.35	1.19	0.24–7.56	0.736
Type of hospitalization (non-ICU = 0, ICU = 1)	3.95	1.895	1.54–10.11	0.004	2.66	1.48	0.89–7.91	0.079
Time from catheter to discharge (2 or more days)	0.98	0.009	0.96–1.00	0.035	0.98	0.01	0.96–1.00	0.085
Previous hospitalizations (no = 0, yes = 1)	1.15	0.825	0.28–4.7	0.846	1.53	1.41	0.25–9.27	0.645
Resistance (no = 0, yes = 1)	2.62	1.322	0.97–7.04	0.057	4.04	2.56	1.17–13.96	0.027

## Data Availability

Data are contained within the article.

## Appendix A. Relationship between Individual Characteristics and Patient Mortality (Time from Catheter to Discharge Categorized), 253 Patients

**Variables**	**Univariate Analysis**				**Multivariate Analysis**			
	**OR**	**EE**	**IC95%**	***p*-Value**	**OR**	**EE**	**IC95%**	***p*-Value**
Sex	0.51	0.15	0.28–0.92	0.025	0.48	0.18271	0.23–1.01	0.055
Age	1.06	0.02	1.02–1.11	0.004	1.07	0.0304	1.01–1.13	0.026
Charlson Category (4 or More)	4.73	3.32	1.19–18.76	0.027	1.86	1.74249	0.29–11.69	0.51
Catheter-Associated Bacteremia	1.8	0.58	0.95–3.39	0.069	4.3	2.48842	1.39–13.37	0.012
Type of Hospitalization	7.52	2.72	3.7–15.29	0	7.09	3.01799	3.08–16.33	0
Previous Hospitalizations	2.14	0.74	1.09–4.22	0.027	1.6	0.74778	0.64–4.00	0.318
Time from Catheter to Discharge								
0 to 6 Days	2.71	1.11	1.21–6.06	0.02	8.4	5.9417	2.1–33.61	0.003
7 to 15 Days	0.8	0.37	0.33–1.96	0.63	3.03	2.21	0.73–12.63	0.128
16 to 34 Days	1.44	0.65	0.6–3.48	0.42	4.03	2.58	1.15–14.13	0.03
35 or More	1	Omitted	--	--	--	--	--	--
								R2 = 0.387
